# Unpacking Constructs: A Network Approach for Studying War Exposure, Daily Stressors and Post-Traumatic Stress Disorder

**DOI:** 10.3389/fpsyg.2015.01896

**Published:** 2015-12-16

**Authors:** Maarten De Schryver, Sofie Vindevogel, Andrew E. Rasmussen, Angélique O. J. Cramer

**Affiliations:** ^1^Department of Experimental-Clinical and Health Psychology, Ghent UniversityGhent, Belgium; ^2^Department of Orthopedagogy, Department of Orthopedagogics and Ghent University College, Ghent UniversityGhent, Belgium; ^3^Department of Psychology, Fordham University, New YorkNY, USA; ^4^Psychological Methods Group, University of AmsterdamAmsterdam, Netherlands

**Keywords:** formative model, reflective model, network model, measurement, PTSD, war

## Abstract

Conflict-affected populations are exposed to stressful events during and after war, and it is well established that both take a substantial toll on individuals’ mental health. Exactly how exposure to events during and after war affect mental health is a topic of considerable debate. Various hypotheses have been put forward on the relation between stressful war exposure (SWE), daily stressors (DS) and the development of post-traumatic stress disorder (PTSD). This paper seeks to contribute to this debate by critically reflecting upon conventional modeling approaches and by advancing an alternative model to studying interrelationships between SWE, DS, and PTSD variables. The network model is proposed as an innovative and comprehensive modeling approach in the field of mental health in the context of war. It involves a conceptualization and representation of variables and relationships that better approach reality, hence improving methodological rigor. It also promises utility in programming and delivering mental health support for war-affected populations.

However, Miller and Rasmussen (2010) show that often a large amount of variance in mental health outcomes remains unexplained, and they point to the role of daily stressors (DS) in understanding mental health in (post-)conflict areas. The potentially predicting, (partial) mediating or moderating role of DS helps to explain the observed differential relations between war exposure and PTSD symptom severity (Barenbaum et al., 2004; Layne et al., 2009; Miller and Rasmussen, 2010). In addition, the stress generation hypothesis has been proposed to argue that DS might not only be a predictor but also a consequence of mental health (Neuner, 2010). For instance, people with psychological distress in the aftermath of war might be stigmatized and excluded, which enhances their likelihood of experiencing stressful daily living conditions. Psychological distress can also influence the appraisal of a traumatogenic event and thus the extent to which it elicits stress reactions. It is not unreasonable to assume that stressful daily living situations also influence exposure to war violence. In the case of child soldiering, for example, social vulnerability often forms the breeding ground for the influx of children into armed groups, which affects their experience with and involvement in war violence (Wessells, 2006). Similarly, PTSD symptoms and DS can also influence the perception and reports of stressful war exposure (SWE), which are typically assessed in a retrospective manner (Karam et al., 1999). This implies that the directly or indirectly defined independent variables or predictors could also be dependent on other variables that have predominantly been considered as dependent variables or outcomes. More specifically, it suggests that variables can often be concurrently considered as outcomes and predictors and that causal loops can exist between these variables.

These recent scientific developments in research on populations exposed to war and other mass casualty events question the traditional reliance on the stress-accumulation theory for modeling stressful events and psychological constructs. We believe they suggest that we need more advanced models that represent the inherent complexity, multiplicity and non-linearity of the relations between SWE, stressful daily living conditions and mental health outcomes. In what follows, we critically discuss the conventional theoretical and psychometric interpretation of the constructs ‘SWE,’ ‘DS,’ and ‘PTSD.’ We then introduce the network approach as an alternative model to investigate the relation between SWE, DS, and PTSD. The overall aim of this paper is threefold. First, we aim to introduce a new perspective into the debate concerning the causal relationships between SWE, DS, and PTSD (for further reading, see Miller and Rasmussen, 2010, and Neuner, 2010). Second, we intend to introduce an innovative methodological approach to the field of mental health in the context of armed conflict. Third, we seek to illustrate how a network model can be created that amalgamates stressful events, both war-related and daily of nature, and post-traumatic stress symptoms.

## The Conventional Modeling Approach: Latent Variable Models

**FIGURE 1:**
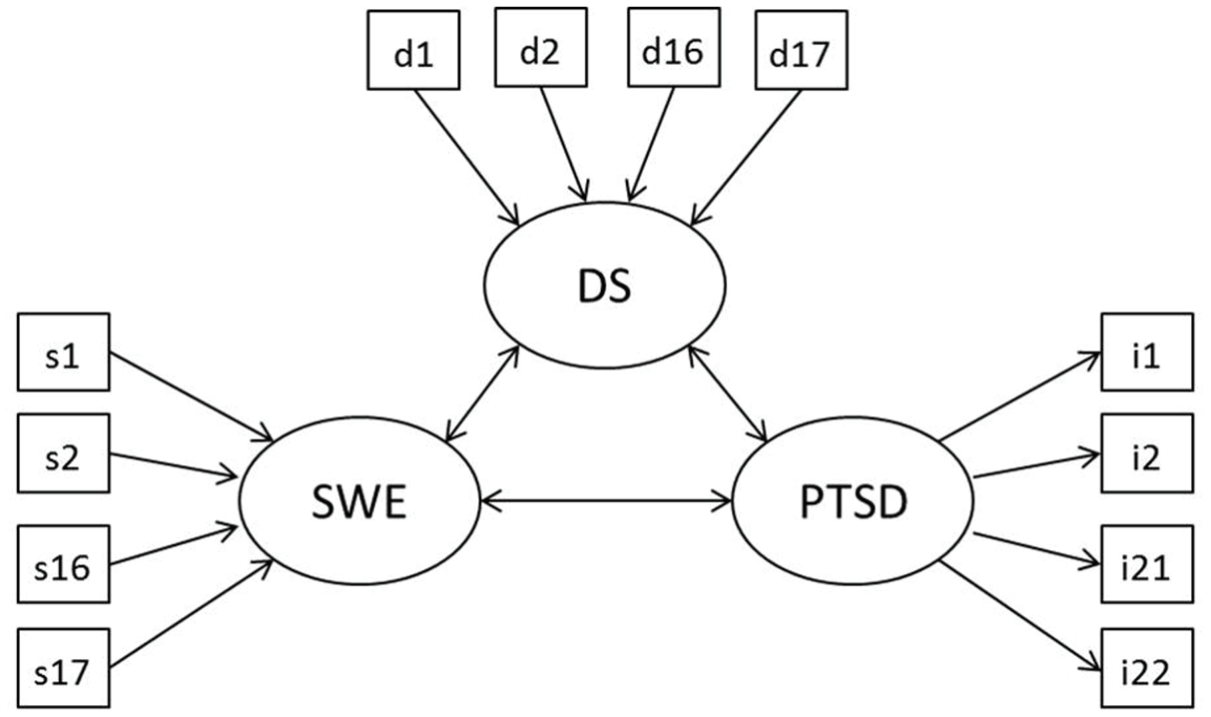
**Structural model defining the relation between SWE, DS and PTSD.** A reflective model is depicted for PTSD, a formative model is depicted for SWE and DS.

**Figure 1** illustrates the structural model often used to define the relation between SWE, DS and PTSD (e.g., in King et al., 1999; Fernando et al., 2010; Dubow et al., 2012; Newnham et al., 2015). Bidirectional arrows represent the possible relations between the constructs. Based on the literature, a direct effect can be expected between SWE and PTSD, between SWE and DS and between DS and PTSD. In accordance with the model proposed by Miller and Rasmussen (2010), DS can serve as a partial mediator between SWE and PTSD. Following the stress generation hypothesis, influence of PTSD on (reported) SWE or DS can be assumed (Neuner, 2010). Moreover, arrows connecting the constructs with their indicators express how these constructs are theoretically defined. Thereby, researchers have -implicitly or explicitly- relied on two measurement models: a reflective (common factor-effect) model and a formative (composite-causal indicator) model (Netland, 2001, 2005; Borsboom et al., 2003; Layne et al., 2009).

If a construct is conceptualized as reflective, the presence of the construct is assumed to be the common cause of the observed variables (i.e., effect indicators). In **Figure 1**, this reflective measurement model is depicted for PTSD, whereby the arrows leading from the PTSD construct to variables i1–i22 indicate a causal effect of the latent construct on the observed variables. Because of their common cause, the variables are expected to correlate and be exchangeable (Schmittmann et al., 2013). If a construct is conceptualized as formative, however, it is regarded as a function of the observed variables (i.e., causal indicators). The co-occurrence of these variables, which can but should not necessarily correlate, is then labeled by a superordinate variable that represents the composite construct. This formative measurement model is depicted in **Figure 1** for the constructs SWE and DS, whereby arrows from the variables s1–s17 and d1–d17 to the constructs SWE and DS, respectively, represent the premise that the single stressful events channel the formation of the stress exposure constructs.

Various authors haven noted that the reflective model has gained most acceptance in psychiatry and psychology (Borsboom et al., 2003; Cramer et al., 2012a) and that it underpins the majority of studies modeling stress exposure and it sequelae in the context of political violence (Netland, 2005). Even though the underlying measurement models are often not explicitly specified, this becomes apparent when looking at the predominant ways in which stressful events and mental health outcomes have been conceptualized and operationalized in the field of armed conflict.

### Stressful Events

While SWE and DS are not usually conceptualized as a latent construct, they are often operationalized as latent variables in the process of measure construction and data analysis. This is demonstrated by Netland (2005), following a critical review of how exposure data in research on political violence are commonly collected and analyzed. Exposure to SWE and DS has most often been measured by means of event lists, such as the War Events Scale (Unger et al., 1998) or Stressful Life Events Rating Scale for Cross Cultural Research (Li et al., 1994). Often, a set of common experiences is selected to represent the stress exposure construct or multiple dimensions of the level of stress exposure (Fernando et al., 2010; Amone-P’Olak et al., 2014; Ertl et al., 2014). Based on the assumption that each stressful event has a similar impact and that the impact of multiple events is additive (cfr. dose-effect relationship), the experience with different SWE or DS is assessed and a cumulative count is calculated as an indicator of stress exposure (Netland, 2005; Jordans et al., 2012; Amone-P’Olak et al., 2014; Ertl et al., 2014). In other studies, a weight is allocated to events with varying degrees of severity and a total exposure score is obtained by adding up the weighted counts (Somasundaram and Sivayokan, 1994; Karam et al., 1999). This aggregated exposure score is then considered as the indication of one’s real stress exposure. Further, common data analytic techniques such as internal-consistency tests (e.g., Unger et al., 1998; Miller et al., 2008; Fernando et al., 2010; Jordans et al., 2012) and factor-analysis for item categorization (e.g., Angel et al., 2001; Steel et al., 2009; Silove et al., 2014) imply a reflective measurement model in which stress exposure is operationalized as a latent variable (Netland, 2005).

Several authors have questioned the underlying causal assumption guiding this conventional way in which event lists are constructed and analyzed, pointing to important theoretical, epistemological and methodological caveats. In response to this, Netland (2005) suggests that, when using a cumulative scale, constructs such as SWE and DS should be treated as composite variables with causal indicators instead. However, when continuing to estimate SWE by latent variables and cumulative counts, certain concerns remain unaddressed. For example, such approaches do not allow one to comprise dimensions such as timing, proximity or severity of stressful events, which might generate an exponential impact and multiplicative effect on mental health in post-conflict contexts (Betancourt et al., 2010). In addition, they do not enable one to explore singular event – symptom relationships, while research is showing that stressful events can impinge on single symptoms or subsets of symptoms, and that the prevalence of these particular symptoms can change over time as a function of changing stressful events (Betancourt et al., 2010; Cramer et al., 2012a). It can therefore be concluded that the use of latent variables and cumulative counts does not allow researchers to discover non-linear or more complex relations between stressful events and mental health outcomes.

### Post-traumatic Stress Disorder

According to the Diagnostic and Statistical Manual of Mental Disorders-IV (DSM-IV)^1^ (American Psychiatric Association [APA], 2000), PTSD is defined in relation to three symptom clusters: avoidance, hyper-arousal and intrusion. Assessing the PTSD syndrome requires all symptom clusters to be present on a clinical level. Mental health outcomes in populations affected by warfare have typically been studied by use of standardized self-report mental health symptom checklists (Layne et al., 2009; Barber, 2013). Such checklists have been developed to assess PTSD (e.g., Impact of Events Scale-Revised), incorporating symptoms that are representative of all symptom clusters (Layne et al., 2009; Rodin and Van Ommeren, 2009). The calculated sum score is usually compared with a cut-off score to determine the clinical significance of the symptoms and to determine the diagnosis of syndromes such as PTSD (Bayer et al., 2007; Layne et al., 2009; Rodin and Van Ommeren, 2009). Typically, factor-analytic techniques are then applied to determine the presence of each symptom cluster (Yule, 1997; Morina et al., 2010, 2011) and internal-consistency is calculated (Bayer et al., 2007; Miller et al., 2008; Fernando et al., 2010; Mels et al., 2010; Ertl et al., 2014).

This practice reflects the underlying view that mental health constructs such as PTSD are latent variables that cause manifest symptoms, which is the basic premise of the reflective model (Borsboom et al., 2003; Schmittmann et al., 2013). Since these latent variables cannot be observed directly, they need to be measured with observable indicators, i.e., the symptoms. As illustrated by Schmittmann et al. (2013), this common practice testifies to the predominant idea in psychology that such measures form a blueprint for actual mental health. In this regard, the obtained symptom sum score represents the extent to which PTSD is actually present and is the common cause of observable symptoms of avoidance, hyper-arousal and intrusion. Following Willemsen et al. (2012), it is reasonable to assume, however, that not everyone develops full PTSD or symptoms representative of all three clusters, but may suffer from a subset of symptoms that nevertheless impede functioning and affect well-being. Since assessments are typically based on a total symptom score, it is likely that this operationalization of PTSD can considerably bias the assessment of clinically significant stress and support needs. For these reasons, the common modeling of mental health outcomes, such as PTSD, as a latent construct has been subjected to criticism (Borsboom et al., 2003; McNally, 2012; Schmittmann et al., 2013).

In conclusion, some important limitations are observed when critically reviewing the common theorizing and study of the relation between stress exposure and mental health in (post-)conflict contexts as well as the modeling of the relation between these constructs and their observed variables. Indeed, the conceptualization of stress exposure and the PTSD syndrome as latent variables, their operationalization by virtue of a selected set of common variables, and their measurement based on aggregated counts risk to mask important ways in which people are differentially exposed to and affected by stressful events (Barenbaum et al., 2004; Netland, 2005; Layne et al., 2009; Betancourt et al., 2010). In what follows, we therefore scrutinize these limitations and introduce the network approach as an alternative way to conceptualize and operationalize stress exposure and mental health in the context of armed conflict.

## Restrictions of Latent Variable Models

It has recently been argued that both the reflective and formative model are limited by their conception of constructs as latent variables and by their representation of a unilinear causal relationship between indicators and constructs (Borsboom et al., 2003; Edwards, 2011; Schmittmann et al., 2013). To begin with, the implicit causality and direction of this causality between indicators and constructs is mostly based on thought experiments and logic instead of scientific evidence (Edwards and Bagozzi, 2000; Schmittmann et al., 2013). For instance, it is questionable whether it is more plausible that the degree of war exposure (construct) causes stressful war-related events (indicators) to occur or whether the occurrence of stressful war-related events (indicators) is constitutive of the composite war exposure (construct). Furthermore, the assumed unilinear relationship between indicators and constructs does not take into account the possibility of cyclic causal trajectories between indicators (Schmittmann et al., 2013). In the context of armed conflict, it is conceivable that, for instance, sickness reduces opportunities to work and generate income, which may result in lack of money, which restricts possibilities to eat a balanced diet and pay for healthcare, which in turn may worsen health conditions and invoke sickness. This example illustrates that DS (indicators) can be caused by other DS (indicators), and that these may even relate in a reciprocally reinforcing manner. As such, indicators rather than the construct seem to play a role in the etiology of other, related indicators (Borsboom and Cramer, 2013). Neither a reflective nor a formative model enable the possibility of such reciprocally influential relations between indicators.

Another restriction of these models is that they limit possibilities to study differential relationships between stressful events and mental health outcomes in various persons. The literature on psychological syndromes such as depression shows growing evidence that stressful events affect singular symptoms and that different types of stressful events are related to different symptom profiles (Keller et al., 2007; Cramer et al., 2012a). This renders it promising to explore the myriad causal pathways between distinct stressful events and stress symptoms. However, reflective and formative measurement models can be considered as arborescent models (Deleuze and Guattari, 1987), which are limited by their monocausal conception of stress and linear connections between stressors and stress reactions. This implies that these models consider the causal pathway between stress exposure and PTSD to be unitary, while empirical findings increasingly suggests it is multipath (Layne et al., 2009). The mental health sequelae of a particular stressful event or set of co-occurring events are not homogeneous, yet have hitherto often been treated as such under a superordinate label, e.g., PTSD. Galatzer-Levy and Bryant (2013) illustrated that the construct PTSD as conceptualized in the DSM-IV can have myriad symptom presentations and almost 80.000 symptom combinations, which are currently understudied. Moreover, in the context of chronic stress exposure, as usually the case during armed conflict, it is often not clear which (types of) stressful events account for the origin and maintenance of PTSD symptoms, but the available evidence suggests that it is multiple rather than single stressful events (Miller and Rasmussen, 2010). Therefore, there is need to further explore differences in the experience with stressful events and mental health symptoms that underlie diagnoses of PTSD in the wake of armed conflict.

Consequently, these models do not allow researchers to study the complexity, multiplicity and non-linearity of the relationships between diverse types of stressors and mental health outcomes, or mutually between stressors and between mental health symptoms, as recommended on the basis of the aforementioned topical scientific developments. These limitations of reflective and formative measurement models indicate areas to be further explored to more accurately study and better understand the myriad potential relations between SWE, DS, and PTSD in (post-)conflict contexts. This challenges the research field to explore models beyond the typical dichotomy of reflective and formative models (Schmittmann et al., 2013). In response to this and inspired by developments in other fields of psychology (Van der Maas et al., 2006; Borsboom et al., 2011; Cramer et al., 2012a; Borsboom and Cramer, 2013; Schmittmann et al., 2013), we introduce the network approach to studying stressful events and mental health in (post-)conflict contexts.

## Unpacking Stressful War Exposure, Daily Stressors and Post-Traumatic Stress Disorder: The Network Approach

Despite the wide application of the network approach in other scientific disciplines such as nature and physics (e.g., Boccaletti et al., 2006), computer science (e.g., Faloutos et al., 1999), biology and medicine (e.g., Vogelstein et al., 2000), social sciences and sociometry (e.g., Weeks et al., 2002)^2^, it has only recently been introduced in the field of psychology as an alternative basis for modeling and measuring psychological constructs (Cramer et al., 2010, 2012b; Schmittmann et al., 2013), including PTSD (McNally, 2012; McNally et al., 2014). It has mainly been proposed as measurement model (i.e., to relate observable variables to the construct) and not as structural model (i.e., to relate constructs to one another). In this paper, we illustrate the value of the network model for studying the relation between the constructs SWE, DS, and PTSD. We opt to do so for various reasons, one being that stressful events make up an integral part of the PTSD construct, as evident in for instance the DSM-IV diagnostic criteria which include ‘the experience of a traumatic event’ (American Psychiatric Association [APA], 2000). Therefore, PTSD cannot be studied and understood in separation from such stressful events. Furthermore, by incorporating the multitude of stressful events that are likely to exert influence on the origin or maintenance of stress symptoms, the manifold relationships between specific stressful events and symptoms can be investigated. This allows us to explore connections between stressful events and symptoms as well as among stressful events and among symptoms, beyond the typical latent variable and linear models that have hitherto dominated this scientific field.

The network approach conceptualizes psychological constructs as networks of related observable variables. The variables are considered to be ‘autonomous causal entities in a network of dynamical systems’ (Schmittmann et al., 2013) and are part of the construct instead of indicators of this construct. The network model hereby abandons the increasingly contested theorem in psychology that psychological phenomena are latent constructs that can be represented by a set of indicators. As such, the variables that are in reflective and formative models typically considered as indicators of a latent construct, are in the network model treated as autonomous entities with causal power.

**FIGURE 2:**
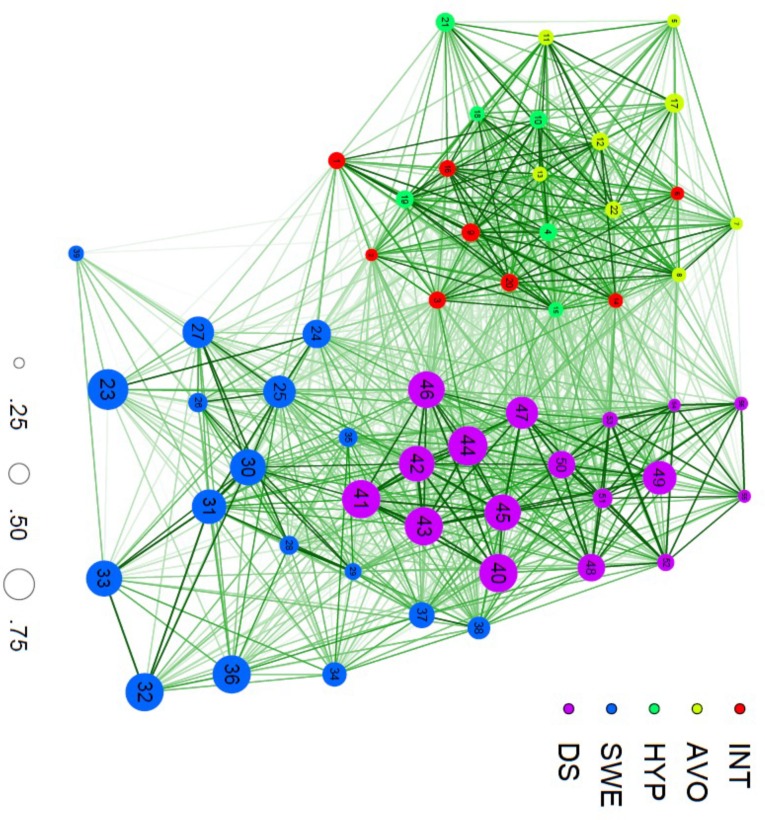
**A network of PTSD, SWE and DS variables.** INT = intrusion, AVO = avoidance, HYP = hyperarousal, SWE = stressful war events, DS = daily stressors. Size of the nodes (0.25–0.75) represents percentage of endorsement of each variable.

**Figure 2** depicts a network created on the basis of a cross-sectional dataset containing information about SWE, DS and PTSD of 445 youths gathered in Northern Uganda. SWE, DS, and PTSD variables were measured with the Stressful War Events Scale (SWE) (Derluyn et al., 2009), Adolescent Complex Emergency Daily Stressors Scale (ACEDSS) (Mels et al., 2010), and Impact of Events Scale-Revised (IES-R)^3^ (Weiss and Marmar, 1997), respectively. The three included constructs can either be interpreted in their own right to detect internal dynamics or be studied in relation to other constructs to search for causal relationships that reveal how they influence one another. The constructs remain clearly identifiable in the network: the variables of PTSD can be found on the left side of the graph, while the right side represents the variables of DS at the top and of SWE at the bottom.

The network was graphically represented by using the R-package ‘qgraph’ (Epskamp et al., 2012). It exists of 56 variables (‘nodes’) and 1540 connectors (‘edges’). Various network analytic techniques have been developed to construe and visualize networks (Boccaletti et al., 2006). In our example, tetrachoric correlation analysis was applied to study strongly correlated sets of SWE, DS, and PTSD variables. These empirical correlations [with correlation or *connectivity* (*r_ij_*)between variables *i* and *j*] resulted in a 56 × 56 adjacency matrix, which served as input for all further network analyses and for the visualization of the network (Borsboom and Cramer, 2013). Average connectivity of the network was 0.28. This measure of network density reflects for this specific structural model the average correlation and indicates that the nodes are not strongly connected.

Next, the nodes’ prominence or *centrality* (the centrality for node *i* is defined as $\frac{\Sigma_{j}r_{ij}}{n}$ , with *n* the number of nodes) in the network was computed based on the mean connectivity of a particular node with other nodes in the network (Boccaletti et al., 2006; Robins, 2013). The nodes most strongly correlated with other nodes can be placed most central in the network (Fruchterman and Reingold, 1991; Schmittmann et al., 2013). In **Figure 2**, the place of nodes in the network thus indicates their centrality and function in relation to the constructs under study. The most central nodes are ‘42. Not enough food’ (0.35; DS), ‘45. Lack of care possibilities’ (0.34; DS), ‘46. Worrying about family’ (0.34; DS), ‘9. Pictures about it popped into my mind’ (0.34; PTSD), and ‘43. Not enough clothing’ (0.33; DS). These central nodes indicate that material, social and institutional loss in the aftermath of warfare plays a central role in the lived experience of these youths.

The *network state* is based on the nodes that are activated in the network; in case of PTSD the variables that represent the actually experienced symptoms. The extent to which the network is activated by the presence and severity of these symptoms forms an indication of the extent to which these symptoms are experienced as clinically significant. In **Figure 2**, the percentage of endorsement of each variable was used as the basis to determine the size of the nodes in the graph visualizing the network. The larger the nodes [e.g., ‘23. Death of loved ones’ (SWE), ‘41. Not being able to pay school fees’ (DS), and ‘44. Sickness in the family’ (DS)], the more they are commonly experienced by the participants.

The *network structure* reflects the strength of the edges between the nodes in the network, by virtue of their number and width. In **Figure 2**, the network generally exists of rather short edges and a high degree of clustering. The stronger the edges between the nodes, the more likely that the activation of one node will activate other, related nodes.

When looking at the correlational structure of the PTSD cluster, for instance, all nodes commingle and the subscales intrusion, hyperarousal and avoidance are undistinguishable. It appears that the subclusters of strongly correlated symptoms do not entirely represent the three PTSD-symptom clusters, because certain symptoms correlate equally or even stronger with symptoms of another cluster. For instance, the hyperarousal node ‘19. Reminders of it caused me to have physical reactions, such as sweating, trouble breathing, nausea, or a pounding heart’ appears to be more and stronger connected with intrusion nodes. This illustrates the value of studying connections at the level of symptoms rather than of latent constructs for obtaining more nuanced insights into stress experiences in (post-)conflict contexts. The graph also shows clustering of DS nodes into two main constellations. The first cluster situated on top of the network (existing of nodes ‘48. Physical punishment,’ ‘49. Others talking ill of you and your family,’ ‘50. Being discriminated against,’ ‘51. Being persecuted by bad spirits,’ ‘52. Abandonment by family,’ ‘53. Abandonment by society,’ ‘54. Forced into marriage,’ ‘55. Do not know my father,’ and ‘56. Disagreement with family’), all refer to familial and social issues in the aftermath of war in northern Uganda. The second constellation located in the center of the network (included nodes ‘40. Feeling of insecurity,’ ‘41. Not being able to pay school fees,’ ‘42. Not enough food,’ ‘43. Not enough clothing,’ ‘44. Sickness in the family,’ ‘45. Lack of care possibilities,’ ‘46. Worrying about family,’ and ‘47. Too much work’) pertain to a living situation characterized by poverty and precarity in the wake of devastating warfare. This may point to reciprocal reinforcement of loss experiences. Such collections of variables can be seen as emerging constructs conceived on the basis of the data (Cramer et al., 2012b). The centrality of the latter cluster and more specifically its location adjacent to SWE suggest that this type of DS is more closely and directly related to experiences with warfare, compared to the second cluster of DS. Furthermore, a modest gap can be identified between the stressful event nodes on the right side and the symptom nodes on the left side of the graph. The network model thus clearly distinguished the stressful events from the symptoms. This implies that the correlations within the cluster of symptom nodes and within the cluster of stressful event nodes respectively were stronger than the correlations between the symptom and stressful event nodes. However, intermediary nodes, situated between the aforementioned clusters, suggest strong connections between stressful events and symptoms.

The state of the network can change qualitatively over time (Schmittmann et al., 2013). This enables researchers to focus on *network dynamics* and allows a processual approach of causal relationships, which is important to collect scientific evidence on causality on the basis of longitudinal research designs. Information concerning the psychological construct under study should be derived from the network state, structure and dynamics (Schmittmann et al., 2013).

## Advantages of a Network Approach For Science and Practice

The network approach enables decomposition of constructs and identified effects, which allows researchers to study and understand differential relations between particular stressors and related mental health symptoms. It is precisely one of the central features of a network approach that makes it possible to visualize how individual networks are differently activated and structured in the wake of particular stressful events or in the presence of certain mental health symptoms. By unpacking the constructs and exploring them at the level of the variables and the relationships between these variables, the network approach has potential to inform the understanding of what it concretely entails for one to have experience with stress exposure or be diagnosed with PTSD. This can significantly further substantial theory building of differential causal effects of stress exposures on mental health in (post-)conflict settings. Moreover, it enables researchers to model relationships among variables, and to include variables concurrently as outcomes and predictors. Patterns of covariance between SWE, DS, and PTSD variables may suggest that particular stressful events evoke particular symptoms and/or that particular symptoms lead one to experience certain stressful events. These are important avenues for future longitudinal network analysis. That would, for instance, enable the investigation of which PTSD-symptoms and associated behaviors evoke stigmatization of former child soldiers, and how the stressful character of such revictimization on a daily basis might reinforce or reactivate PTSD-symptoms –thereby providing valuable insight into regularly observed causal loops between war events, stigmatization and PTSD in (post-)conflict contexts (Neuner, 2010). Such a network model involves a conceptualization and representation of variables and relationships that better approach reality and represent the actual research context, hence improving methodological rigor.

Besides the scientific advantages it holds, a network approach promises utility in programming and delivering support for war-affected populations. As the stressful events with highest centrality and strongest correlations are indicative of common co-occurrence and possibly of excitatory effects toward other events, this result of the network analysis may be informative for preventing a deteriorating chain of stressful events to develop during and after war. Moreover, it may help to address specific mental health needs in relation to specific types of stressors. Knowledge on the centrality of stressful events and symptoms and of patterns of covariance is valuable for preventive interventions, since it shows which stressful events or symptoms are connected with many other symptoms and thus have the potential to incite these and deteriorate one’s mental health. Besides, networks can also be created for individuals, for instance based on time-series data in experience-sampling studies (Borsboom and Cramer, 2013; Bringmann et al., 2013). Such individual network analysis would allow to estimate intra-individual network parameters (Wichers et al., 2015). It can reveal how people react differently to specific events, which events play a role in the emergence of certain stress symptoms in different people, and which events and symptoms are in dynamic interplay affecting one another. Once such information becomes available, interventions can be tailored to individual experiences and professionals can work directly toward the specific stressful events or symptoms. This implies that leaving out the latent syndrome leads to a more nuanced image of the mental health of war-affected people. Moreover, it shows where modification of the causal relation between and among these specific stressful events and mental health symptoms is likely to effectuate a change. It thus reveals possible intervention foci and indicates which particular sources of distress should be tackled to impact on the symptoms. Such information on specific event-symptom connections is hard to obtain from reflective and formative models. The findings obtained through network analysis may thus inform mental health services and psychosocial interventions, to better achieve their goal of delivering customized care to the diverse population of people living in war zones. When central stressful events and symptoms have been tackled properly, it is possible to evaluate how the structure and state of the network alter as a result of this intervention.

## Discussion

In the context of armed conflict, where exposure to war violence and demanding living conditions is omnipresent, people are found to respond differently to these experiences in terms of mental health outcomes (Attanayake et al., 2009). In order to explore this variance, the constructs SWE, DS, and PTSD have been studied extensively. Hereto, researchers have mainly relied on the reflective measurement model, which is characterized by the conception of these constructs as latent variables (Netland, 2005). Based on the assumption of a dose-effect relationship, these constructs have also been combined into structural models to study the impact of stress accumulation on PTSD. However, debate surrounds the question how stress exposure and mental health of war-affected populations are related and how their differential relations can best be modeled and studied (Miller and Rasmussen, 2010; Neuner, 2010). Indeed, following emerging etiological insights and findings in the concerned research field, the use of latent variables and the premise of a linear relationship between these variables can be challenged. The aim of this study was to contribute to this debate by advancing the modeling of SWE, DS, and PTSD. For this purpose, the network model was introduced as an alternative, innovative approach in the field of mental health in the context of armed conflict.

To illustrate this, the network approach has been applied to a sample data set of SWE, DS, and PTSD experienced by war-affected youth in northern Uganda. Before discussing how the network approach offers a valuable alternative for studying the relation between these constructs, some potential limitations of the illustration are reviewed. To begin with, the variables (events or symptoms) and the relationships between the variables were measured in one way, implying that the findings are based on single correlations. In future research, the network model could be extended by creating composite variables that are measured in a triangulated way and built on the basis of multiple data (e.g., nightmares: diary, clinical interview, observation) in order to obtain stronger indications of the identified correlations (Cramer et al., 2010). Moreover, repeated study of the network could lead to the identification of rather stable collections of variables, which forms another way of strengthening evidence on the particular relationships between variables in the network. Such repeated studies within and between populations reduce the risk of creating sample-specific network solutions. Another restriction of this illustration is the cross-sectional nature of the dataset, which prevents the exploration of causal relationships between the variables and of how the relation between stressful events and symptoms changes across time. To study causal relations between variables, one could design longitudinal studies and use, for instance, regression coefficients as input for the network (Borsboom and Cramer, 2013). This could be performed at sample level, whereby between-subject findings provide insight on common relations and pathways between items, or on an individual level to study within-subjects patterns and evolutions (Borsboom and Cramer, 2013). Against the backdrop of the aforementioned limitations of the data set, the network analysis broached preliminary insights and hypotheses that through persistent network analysis have potential to advance the understanding of differential relationships between stress exposure and mental health of youths in this context.

A major advantage of the network model and its move away from latent constructs is that it focuses on the study of empirically identified variables and connections between these variables (Borsboom and Cramer, 2013). Because the models are mainly empirically derived, their generalization should be further explored both within and between different populations. By using a network model to study SWE, DS, and PTSD, one can explore various combinations of symptoms, account for cyclic causal trajectories between stressful events, and explore various paths between SWE, DS, and PTSD variables. It thus considerably advances the existing modeling possibilities (Borsboom and Cramer, 2013). Another advantageous feature of the network model is the use of new measures such as centrality; from which it can be derived what nodes are more strongly and weakly related to other nodes in the network (Cramer et al., 2012b). Moreover, the visualization of relationships and clusters easily reveals patterns in the data and facilitates a straightforward interpretation, compared with other statistical methods. The visualized network in this paper contains 1540 correlations, yet the major findings are directly derivable from the graph. One can quickly see the nodes that are mostly observed (size of the node), prominent (central position of the node) and strongly connected with other nodes (number and width of edges). What’s more, this network approach adds complexity to the measurement and thereby does better justice to the complex reality in which war-affected populations deal with significant distress. It therefore holds great potential to further investigate the imperative question how stressful events and people’s mental health are related in the context of significant adversity such as war.

## Conflict of Interest Statement

The authors declare that the research was conducted in the absence of any commercial or financial relationships that could be construed as a potential conflict of interest.

## Footnotes

1 Although the fifth edition of the DSM has been released in 2013, the DSM-IV is here referred to because the Impact of Events Scale-Revised –used in the illustration to assess PTSD– was developed in accordance with the DSM-IV symptoms and symptom clusters for PTSD.

2 For more information on the historical development of these applications, we refer to (Kolaczyk, 2009; Barabási, 2011). More information regarding the underlying ideas, methods and measures can be found in Borsboom and Cramer (2013), Robins (2013) and Costantini et al. (2014).

3 These data were dichotomized (‘not at all,’ ‘a little bit’ and ‘moderately’ = 0, ‘quite a bit’ and ‘very much’ = 1). Although the practice of dichotomizing continuous variables might cause undesired effects (e.g., MacCallum et al., 2002), we only dichotomize here to facilitate the analyses and interpretation of our example. Note that our example is just for illustrating the major concepts of the network approach, without making any strong empirical conclusions.
